# Intrahepatic Vascular Anatomy in Rats and Mice—Variations and Surgical Implications

**DOI:** 10.1371/journal.pone.0141798

**Published:** 2015-11-30

**Authors:** Constanze Sänger, Andrea Schenk, Lars Ole Schwen, Lei Wang, Felix Gremse, Sara Zafarnia, Fabian Kiessling, Chichi Xie, Weiwei Wei, Beate Richter, Olaf Dirsch, Uta Dahmen

**Affiliations:** 1 Universitätsklinikum Jena, Klinik für Allgemein-, Viszeral- und Gefäßchirurgie, Experimentelle Transplantationschirurgie, Jena, Germany; 2 Fraunhofer Institute for Medical Image Computing MEVIS, Bremen, Germany; 3 Universitätsklinikum RHTW Aachen, Department of Experimental Molecular Imaging (ExMI), Aachen, Germany; 4 Klinikum Chemnitz gGmbH, Institut für Pathologie, Chemnitz, Germany; IDIBAPS - Hospital Clinic de Barcelona, SPAIN

## Abstract

**Introduction:**

The intra-hepatic vascular anatomy in rodents, its variations and corresponding supplying and draining territories in respect to the lobar structure of the liver have not been described. We performed a detailed anatomical imaging study in rats and mice to allow for further refinement of experimental surgical approaches.

**Methods:**

LEWIS-Rats and C57Bl/6N-Mice were subjected to ex-vivo imaging using μCT. The image data were used for semi-automated segmentation to extract the hepatic vascular tree as prerequisite for 3D visualization. The underlying vascular anatomy was reconstructed, analysed and used for determining hepatic vascular territories.

**Results:**

The four major liver lobes have their own lobar portal supply and hepatic drainage territories. In contrast, the paracaval liver is supplied by various small branches from right and caudate portal veins and drains directly into the vena cava. Variations in hepatic vascular anatomy were observed in terms of branching pattern and distance of branches to each other. The portal vein anatomy is more variable than the hepatic vein anatomy. Surgically relevant variations were primarily observed in portal venous supply.

**Conclusions:**

For the first time the key variations of intrahepatic vascular anatomy in mice and rats and their surgical implications were described. We showed that lobar borders of the liver do not always match vascular territorial borders. These findings are of importance for the design of new surgical procedures and for understanding eventual complications following hepatic surgery.

## Introduction

Development of advanced clinical hepatobiliary surgical procedures is closely related to imaging technologies. Advanced imaging allows a precise assessment of the individual intrahepatic vascular anatomy based on 3D visualization prior to surgery. One example for such a development is the novel two stage liver resection techniques called Associating Liver Partition and Portal vein Ligation for Staged hepatectomy (ALPPS), which was introduced in 2012 by Schnitzbauer [1]. This procedure consists of transection of the liver in combination with portal vein ligation prior to liver resection. ALPPS can only be performed on the basis of a sound preoperative visualization of the underlying individual hepatic vascular anatomy of the individual patient.

The progress in clinical hepatobiliary surgery calls for the development of similar procedures in experimental surgery. Rodent models of advanced hepatobiliary procedures may help to better assess the physiological consequences of the newly developed procedures.

In rodents anatomical knowledge is currently limited to the lobar structure of the liver and the basic extrahepatic und intrahepatic vascular anatomy. To our knowledge, the detailed intrahepatic portal vein and hepatic vein vascular anatomy of the four liver lobes, their variations and the corresponding supplying and draining territories in rodents have not been described before.

We performed an exploratory anatomical image study in rats and mice to visualize intrahepatic vascular anatomy and to identify key anatomical variants of potential relevance for experimental hepatic surgery. We used μCT scans for 3D reconstruction and visualization of the intrahepatic vascular trees and the depending hepatic vascular territories. First, portal vein and hepatic vein anatomy was compared to hepatic lobes. Second, we determined the dependent vascular territories and compared them to the underlying lobar anatomy. Potential consequences for hepatic experimental surgery were considered.

## Materials and Methods

### Experimental design

Explanted livers from 22 LEWIS-rats and 26 C57Bl/6N-mice were subjected to μCT-imaging after contrasting the vascular tree with either corrosion cast material (7 rats) or silicone polymer (15 rats, all 26 mice).

The image data were used for semi-automated segmentation to extract the hepatic vascular trees as prerequisite for 3D visualization. The underlying vascular anatomy was analysed and used for determining shape and volume of the dependent territories.

### Animals

Animal experiments were performed using 22 male inbred LEWIS-Rats (Charles River, Germany) with a weight range of 250g to 400g. Furthermore, 26 C57Bl/6N mice (Charles River, Germany)) with a body weight range of 20g to 25g were used. Animals were fed a laboratory diet with water and chow ad libitum and were kept under constant environmental conditions with a 12h light-dark cycle. All procedures, experiments and housing of the animals were carried out according to current German regulations and guidelines for animal welfare and to international principles of laboratory animal care. The protocols were approved by the Thüringer Landesamt für Verbraucherschutz, Thuringia, Germany (approval number: Reg.-Nr. 02-042/10).

### Imaging procedures

#### Contrasting procedure of the vascular tree

All procedures in living animals were performed under inhalation of 1.75–2% isoflurane mixed with an oxygen flow of 0.5–2 L/min (Sigma Delta Isoflurane Vaporizer, PENLON, USA). Intrahepatic vessels were contrasted by injection of Microfil^®^ (Flow Tech Inc., USA), a radiopaque silicon polymer rubber. Alternatively, Batson’s Nr. 17^®^ (Polyscience, Inc., USA), a monomer mixture was used for corrosion casting. Contrast solutions (7mL for rats and 4mL for mice) were prepared according to the corresponding manufacturer’s instructions within 5 min before injection.

Animals were subjected to a laparotomy to have access to the portal vein respectively the inferior vena cava. Vascular access was ensured by placing a catheter either in the inferior vena cava or/and the portal vein.

Injection was performed under visual control. Injection was stopped when the contrast medium appeared on the surface of the organ or in the dissected suprahepatic cava, respectively. Depending on the body and liver weight, a volume of 4–6 mL was needed in rats, respectively of 1.0–1.5 mL in mice. The organs were left in-situ for another 2 hours until polymerization was completed to preserve organ shape. Injected organs were placed in 4% formalin for fixation until scanning took place.

In the rat three portal veins and four hepatic veins were filled with Batson’s Nr. 17^®^ (Polyscience, Inc., USA). Additional four portal veins were injected with Microfil^®^ (Flow Tech Inc., USA). In other 11 rats portal and hepatic vein trees were filled simultaneously.

Furthermore 12 portal veins and 14 hepatic veins of mice were filled with Microfil^®^.

For details see Supplementary Information (S1 Table).

#### Ex vivo micro-CT

The explanted livers were subjected to μCT scanning using TomoScope^®^ DUO (CT-Imaging, Germany) and the scan-protocol HQD-6565-360-90. Dual-sources of the μCT were run with a voltage of 65 kV and a current of 0.5 mA, and 720 projections in one revolution (360°) were acquired with 1032 × 1012 pixels over 90 seconds. Ex vivo μCT resulted in a resolution of 70μm in rat and mouse livers.

### Image Analysis

Image quality was evaluated by assessing the branching level of the vascular tree and by determining the vascular diameters. Visualization of hepatic territories was based on 3D reconstructions with a high resolution defined as a minimal vascular diameter of 70 μm and a branching level of 3rd order.

3D μCT-data was preprocessed by applying a filter for noise reduction and background compensation [2]. The relevant vascular structures (portal and venous system) were segmented by an extended region-growing algorithm [3] or a new semiautomatic vessel-specific segmentation algorithm. Intrahepatic vessels were automatically converted to a hierarchical graph representation. Major branches and branches of subtrees were identified from this hierarchical model and labelled with different colours during exploration of the 3D portal venous and hepatic venous graph. A fusion of the results of vascular analysis and liver segmentation for the volume calculation of individual vascular territories was enabled by the use of mathematical models [4]. The term “territory” refers to the part of the liver that is supplied by a given portal vein branch or drained by a given hepatic vein branch and represents an individual functional unit [5].

## Results

First, we analysed portal vein and hepatic vein anatomy with respect to the liver lobes. Second, we determined the vascular territories and related them to the underlying lobar anatomy.

### Portal vein anatomy and anatomical variations

**Fig 1:**
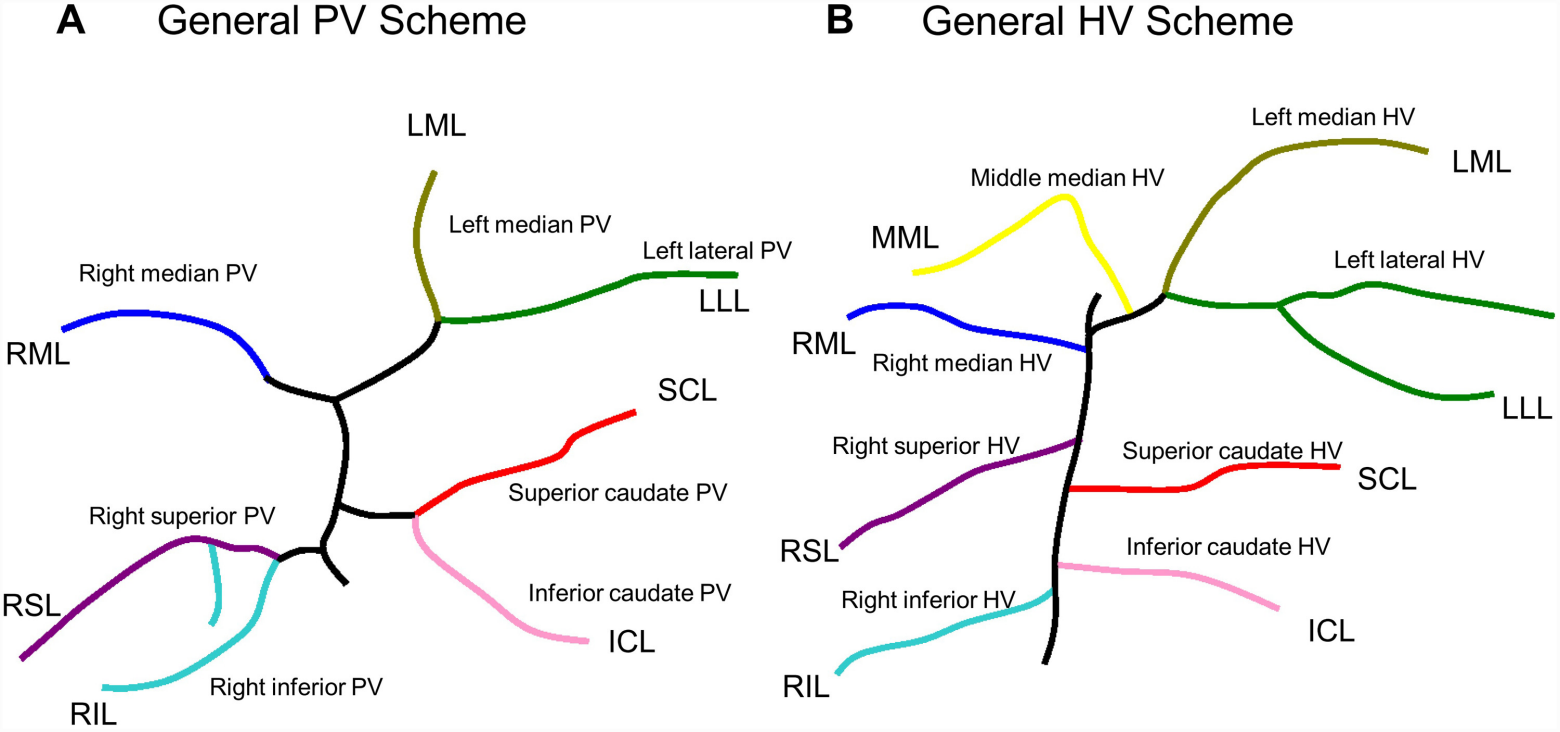
General hepatic vascular anatomy in rats (schematic drawing). **A)** General scheme of portal vein anatomy. **B)** General scheme of hepatic vein anatomy. HV-Hepatic vein, ICL-inferior caudate lobe, MML-middle median lobe, LML-left median lobe, LLL-left lateral lobe, PV-portal vein, RIL-right inferior lobe, RML-right median lobe, RSL-right superior lobe, SCL-superior caudate lobe.

To facilitate the understanding of the complex intrahepatic vascular anatomy we briefly summarize the known basic findings: The portal vein collects the blood from the mesenteric veins and forms the portal vein stem. The portal vein stem divides into two main branches, the right median portal vein supplying the right median lobe and the left portal vein supplying the left median lobe, the left lateral lobe (Fig 1A). In general, the portal supply for the right and the caudate lobes branches off the main portal stem below the main bifurcation.

#### Portal vein anatomy and surgical implications in rats

We analyzed portal venous vascular trees of 18 rats, 3 derived from corrosion casting with Batson#17 and 15 from Microfil^®^ injected samples. In 11 animals both vascular trees were contrasted simultaneously, see also supplementary information (S1 Table). The anatomical variations and their surgical relevant implications are described in the text below and in Table 1.

**Table 1:** Portal vein variations with surgical implications in rats.

Liver lobe	Portal vein variation	Surgical implication
**Right lobe**	right superior PV supplying only the RSL	ligation of RL and separate resection of RIL or RSL is possible
	right superior PV providing one additional caudal or two additional branches of second order into the RIL	resection of RSL with ligation of the right portal vein will cause a lack of portal/arterial supply in half of the RIL leading to a reduction of functional liver mass
	right superior PV providing one posterior branch to the right paracaval liver	ligation of the right portal vein prior to resecting of the RL will cause atrophy/necrosis of the right half of the paracaval liver leading to a reduction of functional liver mass
**Median and left lateral lobe**	substantial distance between bifurcation of left portal stem and left median portal vein and bifurcation of left portal vein and left lateral portal vein	isolated resection is possible
	minor distance between bifurcation of left portal stem and left median portal vein and bifurcation of left portal vein and left lateral portal vein	isolated clamping deep in the parenchyma of left lateral lobe is necessary when resecting LLL or LML
	(lleft median and left lateral portal almost forming trifurcation with left portal vein stem) (Fig 1A)	high risk of compromising the supply of the LML
**Caudate lobe**	one caudate portal vein stem dividing in two main branches of second order	ligation of caudate portal vein with or without hepatic artery will lead to atrophy/necrosis of the left half of the paracaval liver
	difference in terms of branching angle and distance of the branches to portal vein stem	ligation of caudate portal vein with or without hepatic artery will lead to atrophy/necrosis of the left half of the paracaval liver
	one portal vein branch of third order supplying the left paracaval liver	ligation of caudate portal vein with or without hepatic artery will lead to atrophy/necrosis of the left half of the paracaval liver

Each liver lobe has its own portal supply, with the exception of the paracaval liver. The paracaval liver is supplied by various branches from the other lobar portal veins, as described later.

**The right lobe** has one portal vein stem, which divides at the common base of the right lobe into a right superior portal vein and right inferior portal vein. In 5 of 18 cases, the right superior portal vein supplied the right superior lobe only. The right superior portal vein provided one additional caudal (6 of 18 cases) or two (7 of 18 cases) additional branches of second order into the right inferior lobe (Fig 1A) and one posterior branch to the right paracaval liver. The latter is surgically relevant. Resection of the right lobe can be performed with or without ligation of the portal triad. Ligation of the right portal vein with or without hepatic artery prior to resecting the right lobe will also cause atrophy/necrosis of the right half of the paracaval liver leading to a reduction of functional liver mass.

**Fig 2:**
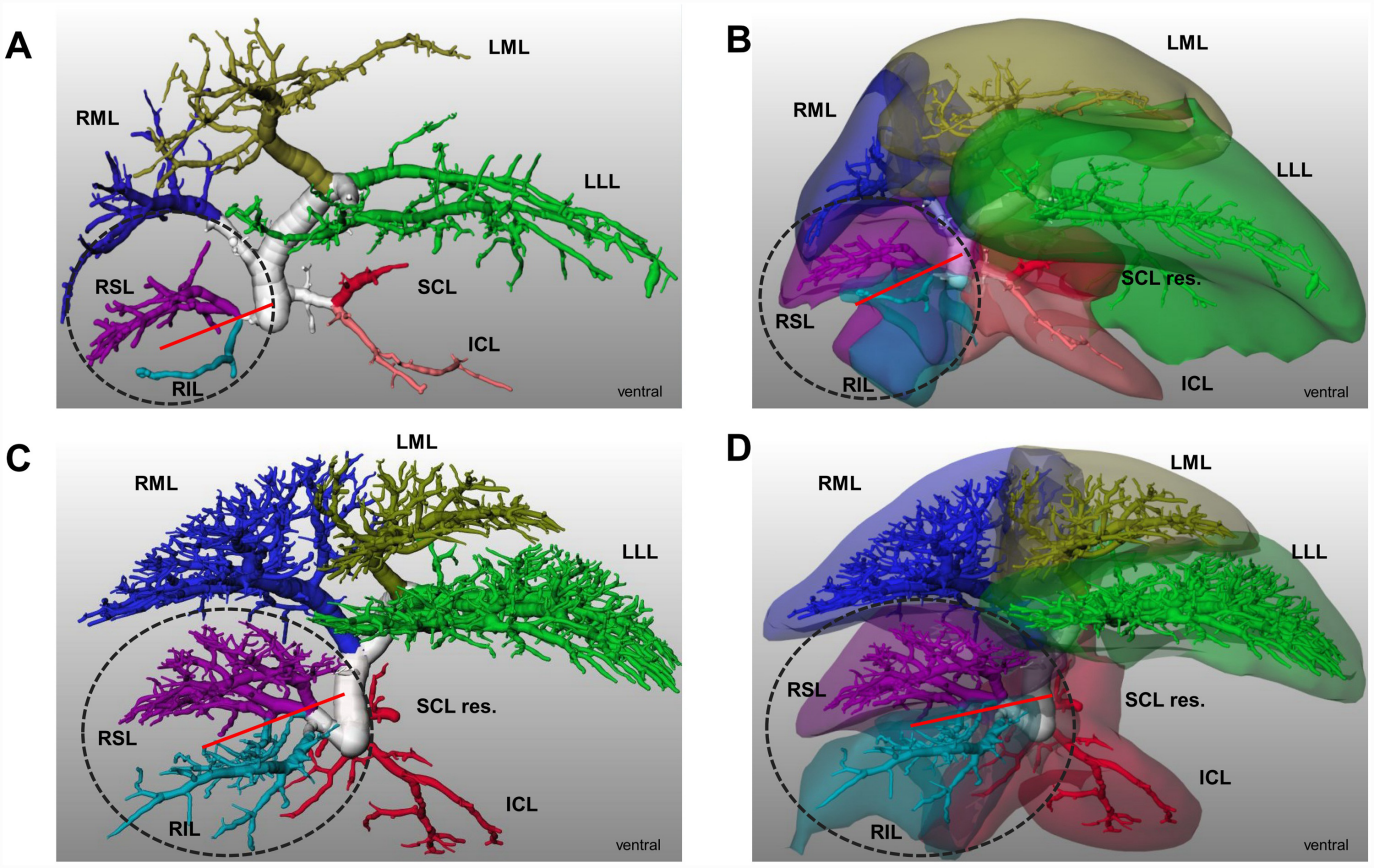
Portal vein variations of the right lobe and surgical implications in rats. 3D visualization. **A)** Visualization of portal vein vascular tree. Single supply of RSL and RIL was observed in 4 of 14 cases. **B)** Portal venous territories. A ligation of right superior PV or resection of RSL without compromising of portal venous supply of RIL is possible. **C)** Portal venous territories. The craniodorsal part of the RIL is supplied by right superior PV in 10 of 14 cases. **D)** Portal venous territories. Ligation of right superior PV or resection of RSL leads to lack of portal supply in half of the right inferior lobe. Red line is resection line or ligation of PV. Encircled is the right lobe. ICL-inferior caudate lobe, LML-left median lobe, LLL-left lateral lobe, PV-portal vein, RIL-right inferior lobe, RML-right median lobe, RSL-right superior lobe, SCL-superior caudate lobe.

The right inferior lobe was supplied either by a single right inferior portal vein branch (6 of 18 cases, see Fig 2A and 2B) or a group of branches (12 of 18 cases). In these 12 of 18 cases the craniodorsal part of the right inferior lobe was additionally supplied by one (6 of 18 cases) or two (6 of 18 cases) branches of third order from the right superior lobe (Fig 2C). The 12 of 18 cases are also of surgical relevance. Resecting the right upper lobe will cause a lack of portal/arterial supply in half of the right inferior lobe, when ligating the right superior portal vein with or without the right hepatic artery prior to resecting the lobe (Fig 2D). This would lead to a necrosis in this area

The **median lobe** and the **lateral lobe** are supplied by the main stem of the portal vein. The right median portal vein (2nd order) supplies the right median lobe.

The left portal vein branches into the **left lateral lobe** and the **left median lobe**. The spatial distance between these bifurcations varied. In 9 of 18 cases there was a substantial distance between branching of left portal vein in left median lobe and left lateral lobe estimated visually.

In the other 9 of 18 cases, left median and left lateral portal vein formed almost a bifurcation deep in the parenchyma of the left lateral lobe (Fig 1A). This is surgically important when resecting the left lateral lobe. In this case the clamp has to be placed rather deep into the parenchyma leaving a large necrotic stump behind to avoid the high risk of compromising the supply of the left median lobe.

The **caudate lobe** has one portal vein stem which divides in two main branches of second order. They supply the inferior caudate and superior caudate lobe (Fig 1A). Individual animals differ in terms of branching angle and distance of the branches to portal vein stem.

One portal vein branch of third order supplies the left paracaval liver. This finding is also of surgical relevance. When resecting the caudate lobe, ligation of portal vein with or without hepatic artery will lead to atrophy/necrosis of the left half of the paracaval liver. This is of importance when performing a subtotal liver resection leaving a small remnant liver behind. In this case the difference in functional liver volume may be decisive for the postoperative outcome with a high risk of postoperative liver failure.

The **paracaval liver** is supplied, as mentioned above, by branches of two different portal veins: the left half by a third order branch of the caudate portal vein, the right half by a second order branch from the right portal vein. This is important, since the paracaval liver may suffer in case of resection of other liver lobes. Compromising the paracaval liver may pass undetected in case of smaller liver resection, but integrity and function of the paracaval liver becomes very important in case of extended liver resection such as 90% partial hepatectomy.

#### Portal vein anatomy and surgical implications in mice

**Fig 3:**
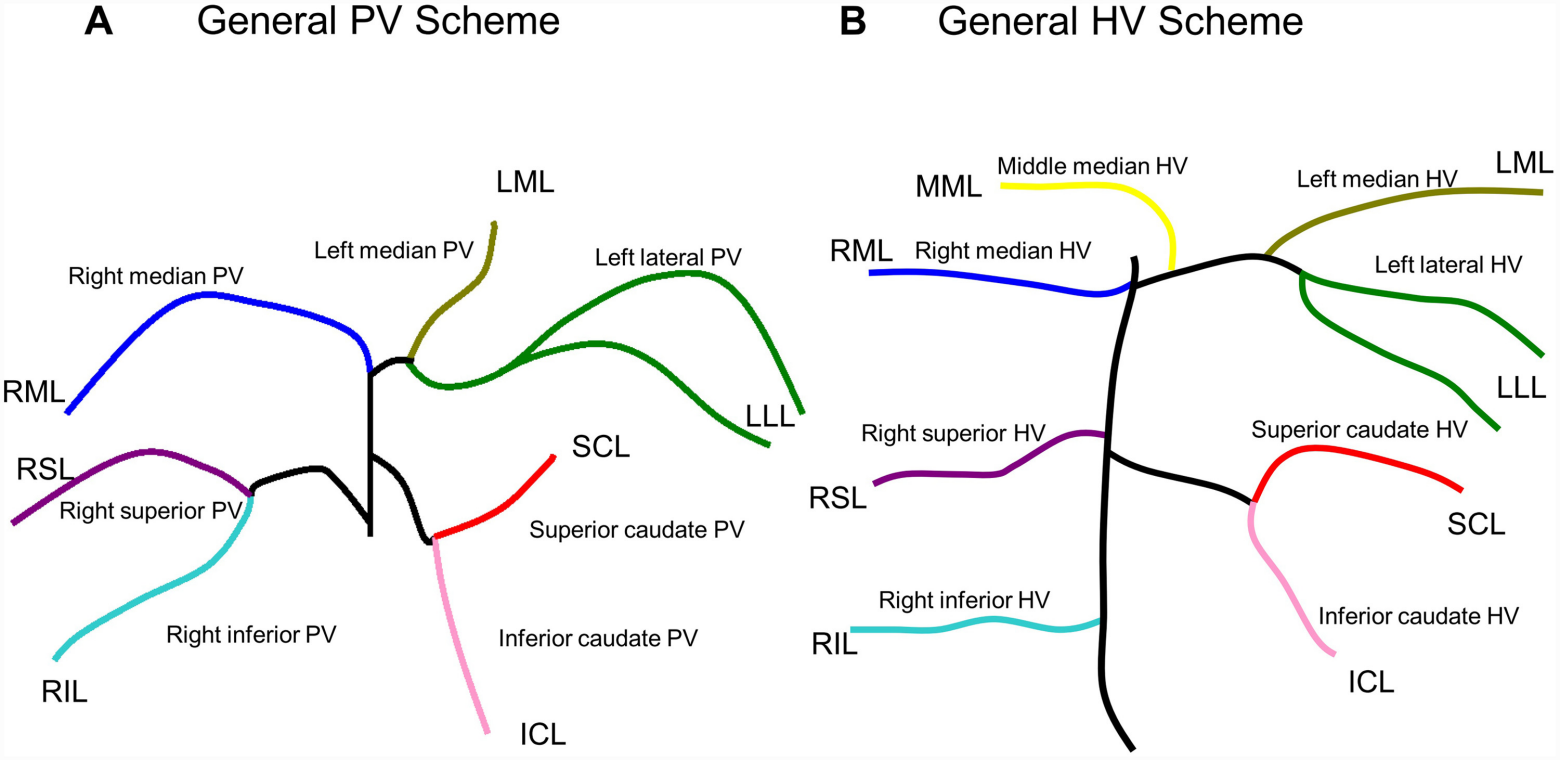
General hepatic vascular anatomy in mice (schematic drawing). **A)** General scheme of portal vein anatomy. **B)** General scheme of hepatic vein anatomy. HV-Hepatic vein, ICL-inferior caudate lobe, MML-middle median lobe, LML-left median lobe, LLL-left lateral lobe, PV-portal vein, RIL-right inferior lobe, RML-right median lobe, RSL-right superior lobe, SCL-superior caudate lobe.

We analyzed portal venous vascular trees of 12 C57Bl/6N mice. Since the lobar structure of the rat and mouse liver is very similar, the basic anatomical pattern of the vascular system is also very similar (Fig 3A). However, upon a detailed anatomical comparison we identified some differences. The anatomical variations and their surgical relevant implications are described in the text below and also in Table 2.

**Table 2:** Portal vein variations and surgical implications in mice.

Liver Lobe	Portal vein variation	Surgical implication
**Right Lobe**	one common right portal vein dividing into a right superior and right inferior portal vein (Fig 3A)	isolated resection of right inferior or right superior lobe is possible
	right superior and right inferior portal vein originating directly from the main portal stem	isolated ligation of right superior or right inferior portal vein ligation is impossible without compromising parenchymal tissue
**Median and left lateral lobe**	one large bifurcation, resulting in a main right portal vein and a main left portal vein	clamping of the main left portal vein prior to resecting the left lateral lobe would result in a lack of supply for the median lobe
	left median and left lateral portal vein (Fig 3A) rising from main left portal vein	isolated clamping of the left lateral portal vein requires a dissection of the left portal vein deep into the hepatic parenchyma
	two right median portal veins forming a trifurcation together with the main left portal vein	two vessels have to be ligated when performing a right median portal vein ligation or resection of the RML
	one portal vein forming a trifurcation together with the left median portal vein and the left lateral portal vein	separate clamping of the two main branches of the left lateral portal vein required prior to a vessel-controlled removal of the left lateral lobe e.g. for 30% or 70% partial hepatectomy

#### Right Lobe

In 11 of 12 cases, the **right lobe** was supplied by a common right portal vein, which divided into an upper right and a lower right portal vein (Fig 3A). In 1 of 12 cases, the right superior and right inferior portal vein originated directly from the main portal stem. This is surgically important when attempting an isolated right superior or right inferior portal vein ligation. In case of this variant isolated ligation of right portal vein is basically impossible without compromising parenchymal tissue.

#### Median and left lateral lobe

In 10 of 12 cases, we observed one large bifurcation, resulting in a main right portal vein and a main left portal vein.

However, we also saw two variations: One case (1 of 12 cases) presented with two **right median** portal veins forming a trifurcation together with the main left portal vein instead of a bifurcation. This anatomical variant has to be considered when planning a right median portal vein ligation, since two vessels have to be ligated.

Another case (1 of 12 cases) presented with a portal trifurcation where the right median portal vein, the left median portal vein and the left lateral portal vein originated from the main portal stem. This is the only situation where removal of the left lateral lobe can be done easily without the risk of compromising the portal supply of the **left median lobe**. In the remaining 10 of 12 cases, the main left portal vein gave rise to the left median and the left lateral portal vein, as already described for the rat (Fig 3A). In these cases, clamping of the main left portal vein prior to resecting the **left lateral lobe** would result in a lack of supply for the median lobe. An isolated clamping of the left lateral portal vein requires a dissection of the left portal vein deep into the hepatic parenchyma.

Additionally, in 1 of these remaining 12 cases described above, vascular anatomy of the left hemi-liver was even more complex. The left median portal vein formed a trifurcation together with the two main branches of the left lateral portal vein. In this case, clamping of the left lateral lobe, e.g., prior to a vessel-controlled removal of the left lateral lobe, would even require separate clamping of the two main branches of the left lateral portal vein.

In all other described cases (11 of 12 cases), the **left lateral lobe** was supplied by the main left lateral portal vein (Fig 3A).

#### Caudate lobe

Three main variations can be distinguished when analysing the portal supply of the **caudate lobe**. The caudate portal vein either originated from the main portal vein stem opposite to the right portal vein (2 of 12 cases) or from the right median portal vein (8 of 12 cases) or from the left portal vein behind the main bifurcation between right median and left portal vein (2 of 12 cases).

### Hepatic vein anatomy and anatomical variations

#### Hepatic vein anatomy and surgical implications in rats

We analyzed hepatic vein vascular trees of 15 rats. Description of hepatic venous drainage also follows the physiological flow direction, starting with the drainage of the caudate lobe.

The **caudate lobe** was drained either by two separate veins (7 of 15 cases) or a confluence of the branches of superior caudate hepatic vein and inferior caudate vein (8 of 15 cases, cf. Fig 2B). In both cases, the territories of the veins corresponded well with the lobar anatomy.

The **right lobe** was always drained by two separate veins (15 of 15 cases, cf. Fig 1B). Territories of the two lobar veins also corresponded well with the lobar anatomy.

**Fig 4:**
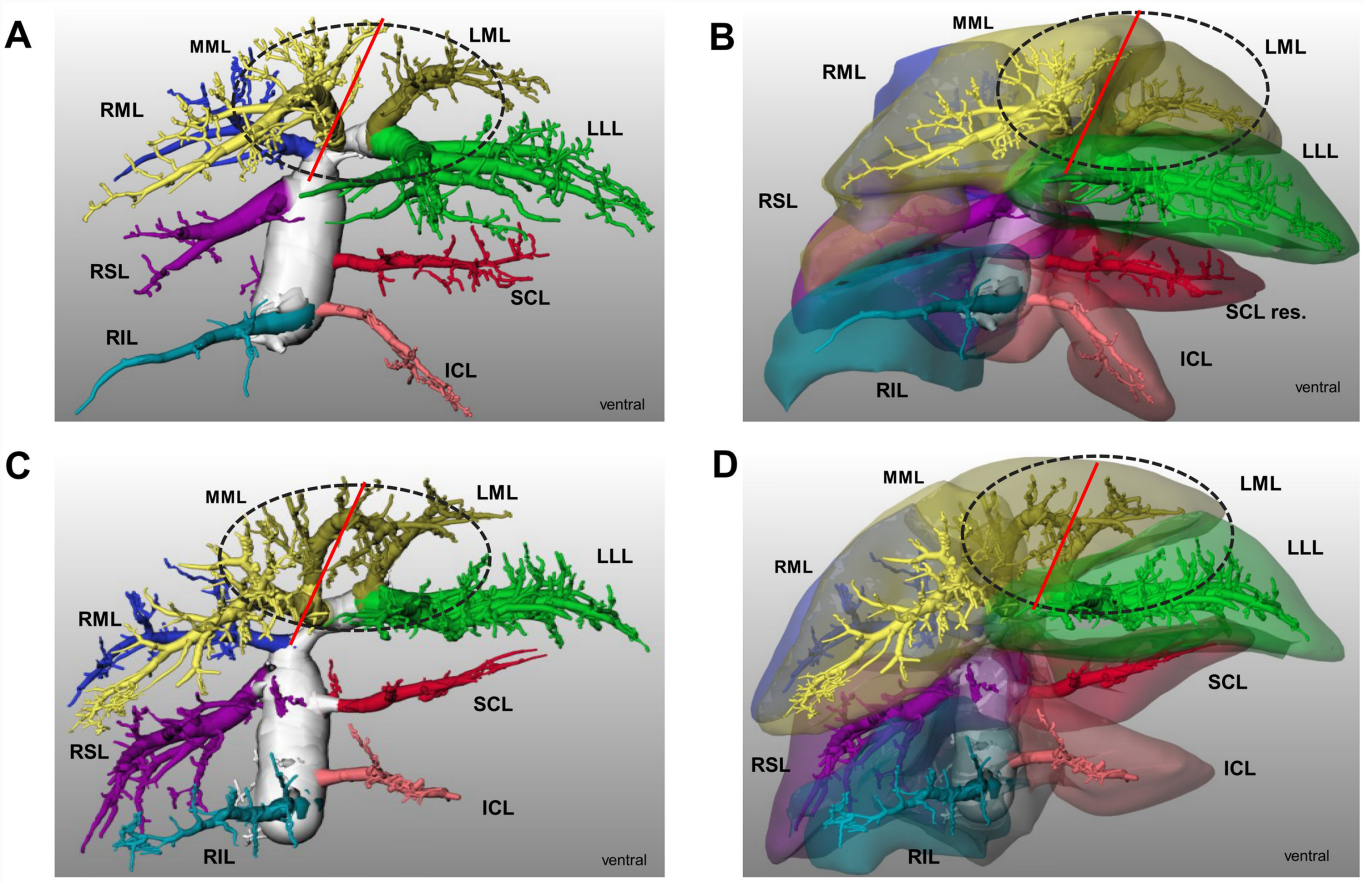
Hepatic vein variations of the right lobe and surgical implications in rats. 3D visualization. **A)** Visualization of hepatic vein vascular tree. One single stem of left median HV was observed in 7/15 cases. **B)** Visualization of hepato venous territories. Transection of median lobe along umbilical fissure with low risk of outflow obstruction of left median lobe possible. **C)** Visualization of hepatic vein vascular tree. Two left median HV stems were observed in 8/15 cases. **D)** Visualization of hepatovenous territories. Transection of median lobe along umbilical fissure with high risk of outflow obstruction of left median lobe. Red line is umbilical transection line. Encircled is the median lobe. HV-Hepatic vein, ICL-inferior caudate lobe, LML-left median lobe, LLL-left lateral lobe, ML-Median lobe, RIL-right inferior lobe, RML-right median lobe, RSL-right superior lobe, SCL-superior caudate lobe.

The **median lobe** was drained by three hepatic veins in all cases (Fig 1B). The territory of the right median hepatic vein was always well defined. The middle median hepatic vein is also anatomically well-defined and drains the middle median sector. The left median hepatic vein showed a higher degree of variability. In 7 of 15 cases we observed a single left median hepatic vein forming a confluence with the branches of left lateral hepatic vein (Fig 4A and 4B). In 8 of 15 cases we observed two branches, one draining into middle median hepatic vein and the other one formed the confluence with left lateral hepatic vein. This is potentially of surgical importance when creating a surgical transection model using the median lobe as applied for revascularisation studies after ALPPS. Transection along the umbilical fissure leaves the right and middle median hepatic vein to the large right “hemi”-liver, whereas the left median hepatic vein ensures the drainage of the small left “hemi”-liver. In case of one left median hepatic vein, the risk of outflow obstruction after transection is low (Fig 4A and 4B). In contrast and in case of two left median hepatic veins the risk for outflow obstruction is rather high (Fig 4C and 4D).

The **left lateral lobe** was primarily drained by a large hepatic vein with 2 to 3 larger branches which formed one large confluence together with the middle median and the left median hepatic vein (Fig 1B).

The **paracaval liver** was drained by multiple small branches directly into the intrahepatic inferior vena cava.

In conclusion, also the hepatic vein system in rats shows considerable variations. However, less surgical implications have to considered compared to the variations in portal vein anatomy.

#### Hepatic vein anatomy and surgical implications in mice

We examined hepatic vein trees of 14 mice. As described above, not only the lobar structure of the rat and mouse liver is very similar, but also the basic anatomical patterns of the vascular systems resemble each other. However, we identified some differences between rats and mice. In the following, we summarised our observation for hepatic veins in mice.

The **caudate lobe** was drained by a confluence of the branches of superior caudate hepatic vein and inferior caudate vein (13 of 14 cases) or by two separate veins (1 of 14 cases). In both cases territories of the veins corresponded well with the lobar anatomy.

The **right lobe** was drained by two separate veins in all cases (14 of 14 cases). Territories of the two right lobar veins corresponded with the lobar anatomy (Fig 3B).

The **median lobe** was drained by three hepatic veins (Fig 3B). The territory of the right median lobe was well defined. The middle median hepatic vein was also anatomically well-defined and drained the middle median sector. In 13 of 14 cases, we observed a single left median hepatic vein forming a confluence with the branches of left lateral hepatic vein. In 1 of 14 cases we observed a variation. The left median lobe was drained by two branches. One branch formed a confluent with the left lateral lobe and one additional branch drained in the middle median lobe.

The **left lateral lobe** was primarily drained by a large hepatic vein which is a confluent of one or two larger branches as observed in the rat. In all 14 cases, the branches also formed one large confluence together with the middle median and left median hepatic vein, leading to the same surgical implication as described for the rat.

The **paracaval liver** was drained by multiple small branches directly into the intrahepatic inferior vena cava.

In conclusion, also the hepatic vein system of mice shows considerable variations, but induced less surgical implications compared to the variations in portal vein anatomy.

## Discussion

The fundamental structure of the four major liver lobes of rat and mouse livers and the segmentation of human liver according to Couinaud [6] is similar and the fundamental structure is comparable [7]. These findings allow the use of rodent models in experimental hepatobiliary surgery.

Detailed knowledge of rodent intrahepatic vascular anatomy and its variation is of importance in experimental hepatobiliary surgery. In clinical surgery this knowledge is needed to develop innovative surgical procedures. Knowledge regarding the individual anatomy of a given patient is also needed in daily surgical routine when performing complex procedures on individual patients. In experimental surgery the knowledge is required to create the corresponding surgical models. Surgical models are the prerequisite for a better assessment of the physiological consequences of newly developed clinical procedures (Table 3).

**Table 3:** Comparison of previous anatomical studies and this study.

Author, year	Species, strain (number)	Technique	Topic and content	Relevance and outcome
**Kogure K, 1999**	Rat, Wistar (n = 20)	In vivo and ex vivo	Description of lobar anatomy of rats and humans	Interpretation of human livers
	Human cadaver livers (n = 78)	Macroscopic anatomical dissection	Description of general extrahepatic and intrahepatic PV and HV vascular anatomy of rats and human	
**Madrahimov N, 2006**	Rat, LEWIS (n = 41)	In vivo resection	Description of Intrahepatic vascular anatomy (HV, PV, HA)	New vessel orientated and parenchyma preserving surgical resection technique for 90%PH
		Ex vivo Corrosion cast	Visualization of vascular supply and drainage	
			Establishment of new resection technique	
**Martins PNA, 2007**	Rat and mouse	Literature review	Description of basic intrahepatic vascular anatomy of HA, PV, biliary system	Rodent models of partial hepatectomies essential tools to study important phenomena in liver research
			Description of topographical liver anatomy	
			Description of different small rodent resection models	
			Description of limitations of rodent resection models	
**Martins PNA, 2007**	Rats, Wistar (n = 12)	In vivo	Description of topographical liver anatomy	Rat and human livers with many similarities but functional anatomical relationships undefined
		Macroscopic anatomical dissection	Description of intra- and extrahepatic vascular anatomy of	
		Literature review	Description of length and diameter of intra- and extrahepatic vasculature	
			Comparison of rat liver and human liver anatomy	
**Fiebig T, 2012**	Mice, C57BL/6 (n = 10)	Literature review	Macroscopic overview over murine liver	Three-dimensional illustrations of macroscopic anatomy of murine liver as reference for future experimental research
		In vivo μCT	Description of lobar anatomy	
			Description of perihepatic structures	
			Description of hepatic vessels	
**This study, 2015**	Rat, LEWIS (n = 22)	Ex vivo μCT of Microfil^®^ sample or corrosion casts of explanted livers	Description of lobar anatomy	First description of intrahepatic vascular variations
	Mouse, C57BL/6N (n = 26)	Ex vivo μCT of Microfil^®^ samples of explanted livers	Description of intra- and extrahepatic vascular anatomy of HV and PV of rats and mice	Definition of hepatic vascular supplying and drainage territories in rats and mice
			Description of hepatic vascular variations of HV and PV of rats and mice	Prerequisite for understanding of surgical complications and estimation of pathophysiological consequences
			Definition of supply and drainage of vascular depending territories	Prerequisite for the development of new experimental hepatobiliary procedures
			Estimation of surgical consequences depending on vascular variations	

Previous anatomical studies described the general lobar anatomy of rodent livers [8, 9]. There are also some studies (see Table 3) describing the basic extra- and intrahepatic vascular anatomy [7–10] but little is known about the variability of the intrahepatic vascular anatomy [9]. Vascular supply and drainage of the single liver lobes were also not yet described in detail [9]. However, this knowledge is important for developing new surgical procedures [8, 10].

Our study closed the existing lack of knowledge concerning variations of hepatic vascular anatomy, the depending vascular territories and their influence on experimental surgical procedures and their consequences (Table 3). To the best of our knowledge, this is the first study of this kind in rodents investigating the intrahepatic vascular anatomy of rats and mice, their variations and their implications for experimental surgery. Furthermore, we assessed the vascular drainage and supply and the depending vascular territories. We pointed out that the vascular territories do not always match the lobar borders, e.g., that the right superior portal vein may also supply the craniodorsal part of the right inferior lobe. The surgical consequences of this finding and of other vascular variations for surgical interventions were described.

To illustrate the relevance of our findings for a better understanding of surgical complications after experimental hepatobiliary procedures, we present one example: Extended 90% liver resection is considered as survival model when following the vessel oriented parenchyma preserving resection technique of Madrahimov (2006) [10]. However, one may encounter difficulties when trying to establish this model. One frequent problem is the unintended ischemic injury to the paracaval liver which may lead to the death of the animal because of an insufficient remnant liver mass [10, 11]. Ischemic injury may occur by ligating the branch to the paracaval liver when placing the piercing sutures in the right superior lobe [10, 11]. Therefore, knowledge of vascular anatomy and variability is necessary, but also knowledge about depending vascular territories to prevent injury of the paracaval liver.

To illustrate the relevance of our finding for clinical surgery, we present another example: Recently, ALPPS was developed as a new procedure to enhance the effect of portal venous deprivation prior to extended liver resection [1]. In contrast to portal vein ligation alone or portal vein embolization, regeneration is highly increased [1, 12]. This effect is explained by the prevention of spontaneous revascularization and portoportal shunt formation when the hepatic parenchyma is surgically transected. However, little is known about the underlying mechanism.

Currently, several research groups including ours are developing a surgical model in the rat to investigate this phenomenon. Like in clinical surgery, the risk for outflow obstruction after transection is directly related to the underlying anatomy of the left median hepatic vein.

Imaging studies are the prerequisite for the development of advanced surgical models. As we show, μCT imaging is very helpful to get precise knowledge of the intrahepatic vascular anatomy and their variations in small rodents. Precise knowledge of possible anatomical variations helps to take these variations into consideration either for developing a new procedure accordingly or for better understanding of anatomically related complications. It remains within the decision of a given experimental surgeon whether to take advantage of this possibility.

## Supporting Information

S1 Table Distribution of animals. Images obtained from different strains of animals were used for the analysis of the hepatic vascular tree. Our goal was to identify key variants with potential surgical relevance. Based on the assumption that visualization of anatomical variants of the major lobar branches is related to the resolution of the imaging technique but independent of the imaging modality, this approach seemed reasonable. Therefore, images from different strains and different casting modalities (S1 Table) were included in the analysis.
(DOCX)

## Abbreviations

μCT: Micro Computed Tomography
3D: three dimensional
ALPPS: Associating Liver Partition and Portal vein ligation for Staged hepatectomy
L: liter
3T: Three Tesla
Gd-EOB-DTPA: gadolinium-ethoxybenzyl-diethylenetriamine penta-acetic acid
mL: milli liter
Fig: Figure
HV: Hepatic vein
ICL: inferior caudate lobe
MML: middle median lobe
LML: left median lobe
LLL: left lateral lobe
PV: portal vein
RIL: right inferior lobe
RML: right median lobe
RSL: right superior lobe
SCL: superior caudate lobe
