# Supplementary material for: Intrahepatic Vascular Anatomy in Rats and Mice—Variations and Surgical Implications
Source: PLoS One. 2015 Nov 30;10(11):e0141798. doi: 10.1371/journal.pone.0141798 (PMC4664386; doi:10.1371/journal.pone.0141798)
Supplement: S1 Table — Images obtained from different strains of animals were used for the analysis of the hepatic vascular tree. Our goal was to identify key variants with potential surgical relevance. Based on the assumption that visualization of anatomical variants of the major lobar branches is related to the resolution of the imaging technique but independent of the imaging modality, this approach seemed reasonable. Therefore, images from different strains and different casting modalities (S1 Table) were included in the analysis. (DOCX) [file pone.0141798.s001.docx]

**Supplementary Information**

**Table S1 Distribution of animals**

Images obtained from different strains of animals were used for the analysis of the hepatic vascular tree. Our goal was to identify key variants with potential surgical relevance.

Based on the assumption that visualization of anatomical variants of the major lobar branches is related to the resolution of the imaging technique but independent of the imaging modality, this approach seemed reasonable.

Therefore images from different strains and different casting modalities (Table S1) were included in the analysis.

|  | **Rat strain** | | | | **Mouse strain** | | | | **SUM** |
| --- | --- | --- | --- | --- | --- | --- | --- | --- | --- |
|  | **LEWIS** | | | | **C57Bl/6N** | | | |  |
|  | **PV** | **PV+HV** | **HV** | **Sum** | **PV** | **PV+HV** | **HV** | **Sum** |  |
| **Ex-vivo µCT: Batson #17** | 3 | 0 | 4 | 7 | 0 | 0 | 0 | 0 | 7 |
| **Ex-vivo µCT: Microfil®** | 4 | 11 | 0 | 15 | 12 | 0 | 14 | 26 | 41 |
| **Sum** | 7 | 11 | 4 | **22** | 12 | 0 | 14 | **26** | **48** |

Literature

[13] [Dahmen U](http://www.ncbi.nlm.nih.gov/pubmed/?term=Dahmen%20U%5BAuthor%5D&cauthor=true&cauthor_uid=18544125), [Radtke A](http://www.ncbi.nlm.nih.gov/pubmed/?term=Radtke%20A%5BAuthor%5D&cauthor=true&cauthor_uid=18544125), [Schröder T](http://www.ncbi.nlm.nih.gov/pubmed/?term=Schr%C3%B6der%20T%5BAuthor%5D&cauthor=true&cauthor_uid=18544125), [Chi H](http://www.ncbi.nlm.nih.gov/pubmed/?term=Chi%20H%5BAuthor%5D&cauthor=true&cauthor_uid=18544125), [Madrahimov N](http://www.ncbi.nlm.nih.gov/pubmed/?term=Madrahimov%20N%5BAuthor%5D&cauthor=true&cauthor_uid=18544125), [Lu M](http://www.ncbi.nlm.nih.gov/pubmed/?term=Lu%20M%5BAuthor%5D&cauthor=true&cauthor_uid=18544125), [Schenk A](http://www.ncbi.nlm.nih.gov/pubmed/?term=Schenk%20A%5BAuthor%5D&cauthor=true&cauthor_uid=18544125), [Peitgen KH](http://www.ncbi.nlm.nih.gov/pubmed/?term=Peitgen%20KH%5BAuthor%5D&cauthor=true&cauthor_uid=18544125), [Dirsch O](http://www.ncbi.nlm.nih.gov/pubmed/?term=Dirsch%20O%5BAuthor%5D&cauthor=true&cauthor_uid=18544125). Median liver lobe of woodchuck as a model to study hepatic outflow obstruction: a pilot study. [Liver Int.](http://www.ncbi.nlm.nih.gov/pubmed/18544125) 2008 Nov;28(9):1236-44.
